# Illness behavior in patients on long-term sick leave due to chronic musculoskeletal pain

**DOI:** 10.3109/17453670902988352

**Published:** 2009-06-01

**Authors:** Patricia Olaya-Contreras, Jorma Styf

**Affiliations:** Department of Orthopedics, Institute of Clinical Sciences, The Sahlgrenska Academy at the University of GothenburgSweden

## Abstract

**Background and purpose** Methods for identification of patients with illness behavior in orthopedic settings are still being debated. The purpose of this study was to test the association between illness behavior, depressed mood, pain intensity, self-rated disability, and clinical status in patients with chronic musculoskeletal pain (CMP).

**Methods** We examined 174 consecutive sick-listed patients (90 women). Musculoskeletal function was estimated by range of motion, muscle strength, and motor and sensory function. The degree of illness behavior was measured by Waddell signs (WS).

**Results** WS were observed in 47/174 (27%) of the patients, 16% of whom manifested excessive illness behaviour. In general, more patients with WS were depressed (OR = 4.4; 95% CI: 1.8–11) and experienced greater pain (OR = 2.9; CI: 1.1–7.7). No abnormal physical function could be observed in two-thirds of the patients. Other predictive factors for manifesting WS at the clinical examinations were longer sick leave and previous full sick leave (p < 0.05).

**Interpretation** Excessive illness behavior is related to psychological distress in patients with CMP and long-term disability. Thus, some patients may also require psychological assessment. Looking for WS during consultation is useful for targeting other factors that may be important in the diagnostic process.

## Introduction

Illness behaviour has been defined as “the discrepancy between the presence of objective somatic pathology and the patient's response to it” (Pilowsky and Spence 1975, Epstein et al. 2006). It has been measured by a standard assessment of behavioral responses to clinical examination: Waddell signs (WS) (Waddell et al. 1980). It is important to identify illness behavior because it has been associated with inferior outcome after treatment (Staal et al. 2003, Eccleston and Crombez 2007).

Chronic musculoskeletal pain (CMP) is not just a physical disease, but an illness in which the physical disorder is combined with many somatic and psychosocial factors (Linton 2000, Foster et al. 2008). The self-report of the patient is one cornerstone of clinical assessment. In many cases, tissue pathology and pathophysiological dysfunction cannot be detected objectively by clinical means. Subjective health complaints are known to be a consequence of persistent stress and several studies have shown that psychosocial factors are important when analyzing pain (Brosschot 2002, Truchon et al. 2008). Psychological factors have been categorized into 3 main types: emotional, cognitive, and behavioral (Macfarlane et al. 2000, Blyth et al. 2007). It is known that a wide range of emotional and cognitive factors are associated with the perception of pain and response to disability (Dickens 2002, Soares et al. 2004).

We studied the third factor, behavior, and tested the association between illness behavior, depressed mood, pain intensity, self-rated disability, and clinical status in patients who were on long-term sick leave due to CMP.

## Patients and methods

### Patients

We examined 174 consecutive patients (90 women), mean age 45 (23–63) years, who had been on sick leave for 19 (3–96) months. 94 of the patients were born in Sweden and 80 were immigrants. They were all referred from the Social Insurance Office to the Diagnostic Center at Lundby Hospital, Gothenburg, Sweden for a thorough orthopedic evaluation, including an assessment of the capacity to work. All patients were invited to participate in the study. We received their verbal consent before the orthopedic examination. Only patients who could read and write Swedish were invited to complete the questionnaires: the Disability Rating Index (DRI) (Sahlén et al. 1994), which was completed by 153 participants, the Beck Depression Inventory (BDI) (Beck et al. 1988), completed by 149, the Patient's Pain Drawing (PPD), completed by 174, and the Verbal Rating Scale (VRS) (Frank et al. 1982), completed by 155. We regarded BDI scores above 18 as a mild form of depressed mood. We chose a BDI cutoff value of 18 points because it is possible to misinterpret an elevated depression score (Williams and Richardson 1993). We excluded one question on sexual activity because we judged it to be less relevant and inappropriate for this group of patients.

Pain intensity was estimated by a Likert scale (VRS): none (0), mild (2.5), moderate (5), severe (7.5), and very severe (10). The patients' pain drawings (PPD) showed the location of their pain. There were no statistically significant differences between the ratio of complete data and missing data for the BDI scores, the DRI scores, the pain intensity, the duration of sick leave, or the degree of capacity to work (Little's MCAR test, p = 0.3). When these variables were compared by sex and origin of the participants, we found no differences between complete data and missing data (MCAR test, p = 0.2).

### Physical function and assessment of physical impairment

Measurements of physical function included range of motion of the cervical and lumbar spine, all major joints of the upper and lower extremities, and all involved joints as indicated by the PPD. Muscle strength in the lower extremities, shoulder, elbow, and wrist joints was assessed manually. Strength of hand grip was measured with a vigorimeter. Muscle volume was measured by noting the circumferences of the upper and lower extremities. Reflexes, motor function, and sensory function were measured by clinical means. All pain locations (as indicated by PPD) were investigated by palpation as part of the clinical evaluation. Most patients had a wide variety of symptoms and all except 1 had more than 2 pain locations. In making a diagnosis, the results of imaging methods were also taken into account. The diagnostic criterion defined in the ICD-10 was used.

*The capacity to work.* The assessment of the capacity to work in Sweden is a standard procedure, the purposes of which are to determine the cause of sick leave, the degree of disability, and the goals for rehabilitation (Hogstedt 2004). The capacity to work is expressed as an index (scale: 0%, 25%, 50%, 75%, or 100%); 100% means that the person is fully fit for work. The index is used by the Social Insurance Office and the healthcare system (www.socialstyrelsen.se). Our study was part of a more extensive study reported to the Social Insurance Office and which was approved by the Swedish Regional Committee of Medical Ethics (Dnr 7-94).

*Illness behavior.* At the present study, illness behaviour was measured through Wadell Signs (WS). Wadell Signs (WS) has been proposed as a tool for the orthopedist to screen psychosocial distress, basically somatization problems in patients with CMP. The following criteria for WS were used in all 174 patients (Main and Waddell 1998, Sobel et al. 2000, Fishbain et al. 2003): (1) complaints of pain on simulated axial loading of the spine; (2) simulated rotation test of the spine, complaints of pain during simulated rotation test; (3) limited straight-leg raising that was increased substantially on distraction; (4) over-reaction to the clinical examination; (5) disproportionate facial or verbal expression to communicate the experience of pain; and (6) sensory loss or weakness that was inconsistent or could not be accounted for by recognized physiologic processes or measurement. This included a more than twofold variation in the vigorimeter test responses or changes in the anatomical area for sensory loss following repeated investigation. Excessive illness behavior was defined as 3 or more WS. Widespread pain was not used as a criterion for illness behavior, as previously recommended (Fishbain et al. 2003). Inter-rater reliability could not be calculated because only 1 physician (JS) performed the evaluation.

### Statistics

The measured variables BDI, DRI, WA, pain intensity, and WS did not have a normal distribution. Thus, we used non-parametric tests: Mann-Whitney test, Kruskall-Wallis median tests, and Spearman correlation coefficients. Categorical data were compared using the chi-squared test. We performed logistic regression analyses (OR, 95% CI) to test the association between WS and BDI, DRI, duration of sick leave, pain intensity at rest, previous capacity to work, and clinical findings, by adjusting for age. The median value was used for ordinal data (BDI, DRI, and intensity of pain) as a cutoff point. We built 2 models. The first included depression, duration of sick leave, and previous capacity to work. The second included duration of sick leave, pain at rest, and self-rated disability instead of depression because of the positive correlation between DRI and BDI. Sickness absence was included as a confounder in the association between WS and distress. All p-values reported are 2-sided and significant at the 5% level (< 0.05).

### Validity of the WS test

The sensitivity, specificity, positive predictor value (PPV), and likelihood ratios (LRs) were calculated using the BDI, the DRI, and the VRS questionnaires as reference standards. The LR tells how much the pretest probability (i.e. the known reference standards) decreases or increases; thus, an LR of 1 or close to it does not change the pretest probability whereas an LR of > 1 increases it (Bhandari and Guyatt 2005).

## Results

### Illness behavior

Waddell signs (WS) were observed in 47/174 (27%) of the patients. 1 or 2 WS were observed in 11%, and 3 to 6 WS in 16%. The mean and median values for WS were 3.4 and 3 (range 1–6). The mean number of WS was 4 in patients who were depressed and 2 for other patients (p < 0.001). The mean values of pain intensity, BDI score, and DRI index were higher for patients with WS than for patients without WS (Table 1).

**Table 1.** Mean values for different variables, broken down according to whether the patient had Waddell signs, in patients with CMP and on long-term sick leave

Variable (range)	With Waddell signs (n = 47)	Without Waddell signs (n = 127)	p-value
Age, years	44	45	0.3 **^a^**
BDI (0–60)	28	18	< 0.001 **^a^**
DRI (0–100)	73	59	< 0.001 **^a^**
Duration of sick leave (3–96 months)	16	21	0.2 **^a^**
Pain at rest (0–10)	7.0	6.0	0.001 **^a^**
Pain at rest, median value	7.5	5.0	0.02 **^b^**

**^a^** Comparison between groups, Mann-Whitney U-test.

**^b^** Median test (n = 174: values can vary for each variable depending on the total of answered questionnaires).

88% of the patients with WS and 71% without WS were unfit for work (i.e. were on full sick leave). A 25–75% capacity to work (partial sick leave) was observed in 12% of the patients with WS, as compared to 30% in patients without WS. The logistic regression analyses showed that patients with previous partial capacity to work (50–75%) were less likely to manifest WS during the orthopedic examination than patients with previous inability to work (adjusted OR = 0.17, 95% CI: 0.35–0.86; p = 0.03). WS was associated with greater ratings (≥ 7.5) of pain intensity at rest (adjusted OR = 2.9, CI: 1.1–7.7; p = 0.02) (Table 3). The Spearman correlation between WS and disability was 0.37 (p < 0.001). It was 0.33 (p < 0.001) between WS and BDI score, and 0.30 (p < 0.001) between WS and pain intensity. We found that the longer the period of sick leave, the greater the likelihood of manifesting WS during the orthopedic consultation, when adjusting for other risk factors (adjusted OR = 0.96, CI: 0.93–0.99; p = 0.04) (Table 3).

**Table 3.** Univariate and multivariate analyses of predictive variables for illness behavior in patients with musculoskeletal pain

Factor	Odds ratio (95% CI) **^c^**	p-value
*Univariate:*		
Depressed mood BDI > 18	3.7 (1.7–8.2)	0.001
BDI ≤ 18 **^a^**	–	
Time of sick leave **^b^**	0.98 (0.95–1.02)	0.1
Degree of capacity to work (previous)		
capacity to work 50–75%	0.26 (0.07–0.90)	0.03
capacity to work 25%	0.56 (0.11–2.8)	0.5
work incapacity **^a^**	–	
Ache at rest, 10 points	8.4 (1.95–37)	0.004
7.5 points	4.1 (1.10–15)	0.04
5.0 points	1.97 (0.5–8.2)	0.4
2.5–0 points **^a^**	–	
DRI > 63 points	1.06 (1.03–1.08)	< 0.001
DRI ≤ 63 **^a^**	–	
*Multivariate:*		
Depressed mood BDI > 18	4.4 (1.8–11)	0.001
BDI ≤ 18 **^a^**	–	
Time of sick leave **^b^**	0.96 (0.93–0.99)	0.04
Degree of capacity to work (previous)		
capacity to work 50–75%	0.17 (0.35–0.86)	0.03
capacity to work 25%	0.62 (0.10–3.8)	0.6
work incapacity **^a^**	–	
Ache at rest, > 7.5 points	2.9 (1.1–7.7)	0.02
≤ 7.5 points **^a^**	–	
DRI > 63 points	1.05 (1.01–1.07)	0.004
DRI ≤ 63 **^a^**	–	

**^a^** Referent category.

**^b^** Assessed as continuous variable.

**^c^** Adjusted for age.

### Depressed mood and self-rated disability associated with WS

71% of the patients with excessive illness behavior had depressed mood (p = 0.003, chi-squared test). The probability of manifesting WS during consultation was 4 times higher in patients with a BDI score of >18 than in patients with a BDI score of less than 18 (adjusted OR = 4.4, CI: 1.8–11; p = 0.001). The disability rating index was also associated with WS. The higher the DRI scores, the greater the probability of manifesting WS during the clinical examination (adjusted OR = 1.05, CI: 1.01–1.07; p = 0.004).

### Pain location and physical function

67% of patients experienced pain in the neck and shoulders, 21% in the lower back, and 12% in other locations. Accompanying back pain was reported in two-thirds of patients who experienced pain in the neck and other locations. There were no differences between the location of pain and WS (p = 0.4, Kruskall-Wallis). All patients reported musculoskeletal pain on palpation. Normal neuromuscular function was observed in 117/174 (67%) of the patients at clinical investigation. The clinical diagnosis for most of the patients was unspecific pain in the neck and back (e.g. M54.1, M54.2, M54.4, and M54.9). Minor impairment was documented in 57/174 (33%). According to imaging, few of our patients had spondylosis in the cervical and/or lumbar spine. None of the patients had clinical signs indicating foraminal stenosis.

### Nationality and sex

More patients 31/80 (39%) with a non-Swedish background manifested WS than Swedish patients 16/94 (17%) (p = 0.002, chi-squared). The mean BDI score for patients with a non-Swedish background was 26 (SD 13), as compared to 17 (SD 11) for Swedish patients (p < 0.001). Moreover, patients with a non-Swedish background rated their pain intensity to be greater than for Swedish patients (median values 7.5 and 5.0, respectively; p = 0.001, median test). There were no associations between WS and sex or age.

### Validity of the WS test

The ability of the WS test (i.e. specificity) to detect those patients without psychological distress was in the range of 84–88%; however, the sensitivity was poor (< 60%). The proportion of correctly screened patients with psychological distress (PPV) was in the range of 72–88% (Table 2).

**Table 2.** Appraisal of validity of the WS test to screen psychological distress using a matrix

Reference standard	WS test sensitivity, %	WS test specificity, %	WS test PPV, %	LR **^a^**	LR **^b^**
Beck depression inventory (BDI)	39	85	72	2.6	0.71
Self-estimate of disability (DRI)	32	88	86	2.7	0.77
Pain intensity rating (VRS)	32	88	88	2.7	0.77

PPV: positive predictive value.

**^a^** LR: likelihood ratio for positive test.

**^b^** LR: likelihood ratio for negative test.

The LRs for positive WS varied from 2.6 to 2.7. We found that patients with higher scores on the DRI (> 63) were almost 3 times more likely to manifest WS than those with scores lower than 63. Patients with higher scores regarding intensity of pain (> 7.5) were more likely to manifest WS (LR = 2.7). Moreover, patients who had depressed mood were almost 3 times more likely to manifest WS than others (Table 2).

## Discussion

### Illness behavior

We found that one quarter of our patients with CMP manifested WS. This is consistent with other studies, which have reported excessive illness behavior in 12–36% of patients with chronic neck pain and low back pain and in up to 50% of subjects undergoing work disability assessment for worker's compensation (Waddell et al. 1980, Bellamy 1997, Sobel et al. 2000). The Waddell Signs (WS) has been found to be unrelated to physical pathology and age (Novy et al. 1998, Fishbain et al. 2003). Conversely, WS has been associated with the physician's judgement of the functional capacity of the patient (Novy et al. 1998, Fishbain et al. 2003). In our study, excessive illness behavior was unrelated to age and does not seem to be related to loss of physical function, but rather to depressed mood, higher self-rated disability, and greater intensity of pain.

Illness behavior does not exclude organic genesis of pain, but indicates distress. 3 or more WS signs may indicate psychosocial issues, pain behavior, or excessive illness behavior (Fishbain et al. 2003). Half of our patients with CMP had depressed mood, two-thirds of whom manifested excessive illness behavior. These patients were depressed, rated their disability higher, and experienced a greater degree of pain. In addition, we found that patients who reported depressed mood (BDI > 18) were almost 4 times more likely to have WS at the time of the orthopedic evaluation. Depression has previously been reported in patients with CMP in the presence of WS (Novy et al. 1998). It has also been documented that symptoms of somatization and illness behavior diminish when patients are treated for pain (Foster et al. 2008). In a review of 61 studies, WS were associated with poorer treatment outcome and greater levels of pain (Fishbain et al. 2003). In another study, it was found that patients who showed excessive illness behavior took longer to return to work (Werneke et al. 1993). We agree with previous reports that WS should not be used as an isolated predictor of the return to work or of the sickness absence (Waddell 2004).

We confirm the association between WS and poorer physical performance, which is consistent with previous research (Novy et al. 1998, Fishbain et al. 2003). Two-thirds of the participants in our study had been on full disability allowance before this evaluation. We found that under circumstances of inability to work, poorer physical performance, and greater pain scores, patients who had been on sick leave longer were more likely to manifest illness behavior. Possible explanations for excessive illness behavior in our series include learned patterns of behavior, effects of cultural differences and social determinants, way of pain communication, compensation issues, iatrogenic factors, and persistent stress, as previous researchers have described (Brosschot 2002, Hobara 2005, Noyes et al. 2005, Simon et al. 2006, Truchon et al. 2008). Furthermore, some people appear to be more sensitive in the presence of persistent stress and may develop mechanisms mediated by sensitization of specific neurons (Ursin and Eriksen 2007, Loeser and Treede 2008). All these factors, and also the patient's expectations, may to some extent affect how the patient reacts at the medical investigation. Moreover, illness behavior may also be a sign of avoidance and kinesiophobia, both of which may lead to passive behavior (Leeuw et al. 2007).

In the present study, the WS test showed high specificity and gave PPV values with acceptable LRs when BDI, DRI, and pain scores were used as reference standards. These subjective scores may reflect psychological distress. We belive that WS may help clinicians to identify psychological distress, which if it is left untreated may impede recovery.

### Limitations of the study

The main limitation of our study is the selection of the participants who were referred by the Social Insurance Office. The Office requested a second evaluation of the patient's capacity to work. Thus, among these patients we could expect a greater prevalence of illness behavior, greater disability, and greater pain intensity than in other clinical settings. Moreover, the direction of the association between WS and the variables under study could not be determined due to the cross-sectional design. Furthermore, the observation of WS does not itself constitute a psychological evaluation. The lack of a reliability test is an important limitation of the present study. Previlously, good reliability and validity of the WS had been reported for predicting psychological problems in patients with chronic pain (Novy et al. 1998). Even so, we believe that the psychometric properties of the WS (as the reliability test performance) may be an important issue in future studies.

### Musculoskeletal pain and physical findings

Sick-listing in itself is ineffective as a treatment for long-lasting back pain and is associated with high costs in Sweden (Hansson and Hansson 2005). There was a substantial discrepancy between self-rated disability and musculoskeletal function in most of our patients at clinical investigation. Illness as a medically unexplained symptom and its associated disability is a common health problem that demands more medical resources than other complaints (Nimnuan et al. 2000, Hiller et al. 2006). In Norway, for instance, more than half of all patients who are certified as being sick are judged on the basis of subjective health complaints (Ursin 1997), and in the UK two-thirds of recipients of incapacity benefits have health-related problems that cannot be explained in purely medical terms (Waddell 2006). CMP causes considerably increased use of health services, absence from work due to sickness, and early retirement (Wallman et al. 2006).

In summary, Illness behavior was found to be closely related to psychological distress in patients with CMP who had been on sick leave for a long time. It was associated with depression, increased experience of pain, and high self-rated disability. Moreover, it was seen more commonly in immigrants. In two-thirds of the patients with CMP, no physical impairment could be detected. These findings support the importance of the association of pain with WS. Thus, in the process of compensating disability due to CMP, one must take into account an approach that also integrates the behavioural risk factors. Consequently, some patients may also require assessment of the behavioural aspects of their pain. Looking for WS during consultation is a useful tool for targeting other factors that may interfere with recovery from CMP.
